# Diaqua­bis­[4-(1*H*-imidazol-2-yl)pyridine-κ*N*]bis­(nitrato-κ*O*)cadmium

**DOI:** 10.1107/S1600536812050908

**Published:** 2012-12-22

**Authors:** Chuan-Yue Zhang, Tao Wang, Chuan-Ming Jin

**Affiliations:** a Hubei Key Laboratory of Pollutant Analysis & Reuse Technology, College of Chemistry and Environmental Engineering, Hubei Normal University, Huangshi 435002, People’s Republic of China

## Abstract

In the title compound, [Cd(NO_3_)_2_(C_8_H_7_N_3_)_2_(H_2_O)_2_], the Cd^II^ cation is situated on an inversion center and is coordinated by the O atoms of two nitrate anions, by the N atoms of two 4-(imidazol-2-yl)pyridine ligands and by two water O atoms in a slightly distorted N_2_O_4_ octa­hedral geometry. The dihedral angle between the imidazole and pyridine rings is 1.6 (2)°. In the crystal, mol­ecules are linked by N—H⋯O, O—H⋯N and O—H⋯O hydrogen bonds, forming a three-dimensional network.

## Related literature

For background to compounds with metal-organic framework (MOF) structures, see: Batten & Robson (1998 ▶); Burrows (2011 ▶); Jin *et al.* (2010 ▶); Tanabe & Cohen (2011 ▶). For the use of *N*,*N*′-type ligands in MOFs, see: Custelcean (2010 ▶); Pschirer *et al.* (2002 ▶). For the structural analysis of an imidazole closely related to the ligand, see: Voss *et al.* (2008 ▶).
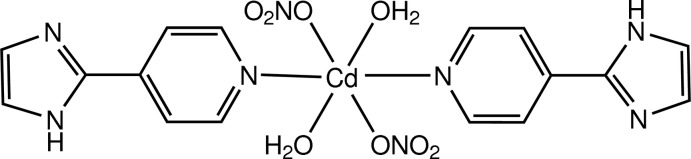


## Experimental

### 

#### Crystal data

[Cd(NO_3_)_2_(C_8_H_7_N_3_)_2_(H_2_O)_2_]
*M* *_r_* = 562.78
Monoclinic, 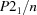

*a* = 7.2508 (7) Å
*b* = 12.1372 (12) Å
*c* = 12.3509 (12) Å
β = 102.278 (2)°
*V* = 1062.07 (18) Å^3^
*Z* = 2
Mo *K*α radiation
μ = 1.09 mm^−1^
*T* = 298 K
0.16 × 0.12 × 0.10 mm

#### Data collection

Bruker APEXII CCD area-detector diffractometer
Absorption correction: multi-scan (*SADABS*; Sheldrick, 1996 ▶) *T*_min_ = 0.845, *T*_max_ = 0.899
6543 measured reflections
2462 independent reflections
2272 reflections with *I* > 2σ(*I*)
*R*_int_ = 0.046

#### Refinement

*R*[*F* ^2^ > 2σ(*F* ^2^)] = 0.035
*wR*(*F* ^2^) = 0.084
*S* = 1.10
2462 reflections
157 parameters
3 restraints
H atoms treated by a mixture of independent and constrained refinement
Δρ_max_ = 0.73 e Å^−3^
Δρ_min_ = −0.47 e Å^−3^

### 

Data collection: *APEX2* (Bruker, 2004 ▶); cell refinement: *SAINT-Plus* (Bruker, 2004 ▶); data reduction: *SAINT-Plus*; program(s) used to solve structure: *SHELXS97* (Sheldrick, 2008 ▶); program(s) used to refine structure: *SHELXL97* (Sheldrick, 2008 ▶); molecular graphics: *SHELXTL* (Sheldrick, 2008 ▶); software used to prepare material for publication: *SHELXTL*.

## Supplementary Material

Crystal structure: contains datablock(s) I, global. DOI: 10.1107/S1600536812050908/im2415sup1.cif

Structure factors: contains datablock(s) I. DOI: 10.1107/S1600536812050908/im2415Isup2.hkl

Additional supplementary materials:  crystallographic information; 3D view; checkCIF report

## Figures and Tables

**Table 1:** Hydrogen-bond geometry (Å, °)

*D*—H⋯*A*	*D*—H	H⋯*A*	*D*⋯*A*	*D*—H⋯*A*
N3—H3⋯O3^i^	0.86	2.10	2.923 (3)	160
O4—H4*B*⋯N2^ii^	0.82 (1)	1.98 (1)	2.796 (3)	174 (4)
O4—H4*A*⋯O2^iii^	0.81 (1)	2.14 (1)	2.946 (4)	174 (4)
O4—H4*A*⋯O3^iii^	0.81 (1)	2.65 (3)	3.197 (3)	126 (3)

Symmetry codes: (i) 
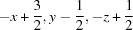
; (ii) 
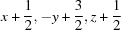
; (iii) 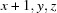.

**Fig. 1.**
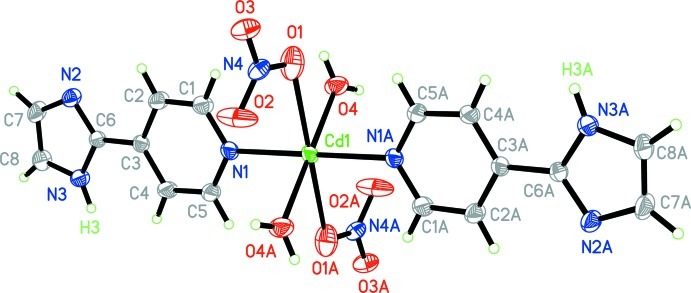
The molecular structure of (I) with atom labels and 30% probability displacement ellipsoids for non-H atoms.

**Fig. 2.**
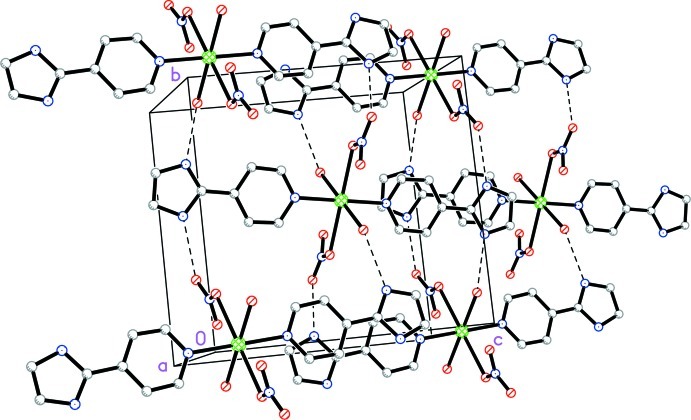
The three-dimensional supramolecular packing architecture of (I) with hydrogen-bonds depicted as dashed lines.

## supplementary crystallographic information

### Comment

The construction of functional metal-organic frameworks is of great interest due to their intriguing network topologies and their potential applications as microporous, magnetism, catalysis, nonlinear optics, molecular separation, toxic materials adsorption and molecular sensors (Batten & Robson, 1998; Burrows, 2011; Jin *et al.*, 2010; Tanabe & Cohen, 2011). The molecular geometry and flexibility of multidentate ligands play key roles in the field of supramolecular self-assemble on metal-organic frameworks. For example, 4, 4'-bipyridine, 1, 2- bis(4-pyridyl)ethane and *trans*-bis(4-pyridyl)ethene as ligands can form a lot of coordination polymers with different structure features (Custelcean, 2010; Pschirer *et al.*, 2002). Our interest is to exploit the coordination chemistry of 2-pyridinyl-imidazole and its derivatives together with their potential application in material science.

In the report, the mono-nuclear cadmium(II) complex, [Cd(C_8_H_7_N_3_)_2_(NO_3_)_2_(H_2_O)_2_], was obtained *via* the reaction of 4-(1*H*-imidazol-2-yl)-pyridine and cadmium(II) nitrate. Single crystal X-ray diffraction analysis reveals that the cadmium(II) atom is six-coordinated in a slightly distorted octahedral geometry by two pyridine nitrogen atoms, two nitrate anions oxygen atoms and two aqua oxygen atoms forming N_2_O_4_ donor set (Figure 1). The cadmium atom is situated on an inversion center. Bond distances of Cd(1)—N(1), Cd(1)—O(1) and Cd(1)—O(4) are 2.276 (2), 2.503 (3) and 2.310 (2) Å, respectivity. The dihedral angle between the imidazole and pyridine rings is 1.6 (2)°. In the crystal, molecules are linked by N—H······O, O—H······N and O—H······O hydrogen bonds, forming a three-dimensional network (Figure 2).

### Experimental

The organic ligand 4-(1*H*-imidazol-2-yl)-pyridine was prepared according to the previously reported literature methods (Voss *et al.*, 2008). Cd(NO_3_)_2_ (24 mg, 0.1 mmol) dissolved in 5 ml ethanol and a solution of 4-(1*H*-imidazol-2-yl)-pyridine (29 mg, 0.2 mmol) in another 5 ml of ethanol were mixed, refluxed for 5 h and filtered. The filtrate was left at room temperature. Suitable single crystals for a X-ray diffraction study were obtained after a few days (yield: 73% based on Cd(II) salts).

### Refinement

H atoms were positioned geometrically at distances of 0.93 (CH), and 0.86 (NH) from the respective parent atoms, a riding model was used during the refinement process. *U*_iso_ values were constrained to be 1.2 times *U*_eq_ of the carrier atom.

### Crystal data

**Table:**

[Cd(NO_3_)_2_(C_8_H_7_N_3_)_2_(H_2_O)_2_]	*F*(000) = 564
*M**_r_* = 562.78	*D*_x_ = 1.760 Mg m^−^^3^
Monoclinic, *P*2_1_/*n*	Mo *K*α radiation, λ = 0.71073 Å
Hall symbol: -P 2yn	Cell parameters from 4257 reflections
*a* = 7.2508 (7) Å	θ = 2.4–28.3°
*b* = 12.1372 (12) Å	µ = 1.09 mm^−^^1^
*c* = 12.3509 (12) Å	*T* = 298 K
β = 102.278 (2)°	Block, colorless
*V* = 1062.07 (18) Å^3^	0.16 × 0.12 × 0.10 mm
*Z* = 2	

### Data collection

**Table:**

Bruker APEXII CCD area-detector diffractometer	2462 independent reflections
Radiation source: fine-focus sealed tube	2272 reflections with *I* > 2σ(*I*)
Graphite monochromator	*R*_int_ = 0.046
phi and ω scans	θ_max_ = 28.0°, θ_min_ = 2.4°
Absorption correction: multi-scan (*SADABS*; Sheldrick, 1996)	*h* = −7→9
*T*_min_ = 0.845, *T*_max_ = 0.899	*k* = −15→15
6543 measured reflections	*l* = −14→16

### Refinement

**Table:**

Refinement on *F*^2^	Primary atom site location: structure-invariant direct methods
Least-squares matrix: full	Secondary atom site location: difference Fourier map
*R*[*F*^2^ > 2σ(*F*^2^)] = 0.035	Hydrogen site location: inferred from neighbouring sites
*wR*(*F*^2^) = 0.084	H atoms treated by a mixture of independent and constrained refinement
*S* = 1.10	*w* = 1/[σ^2^(*F*_o_^2^) + (0.0252*P*)^2^ + 1.0694*P*] where *P* = (*F*_o_^2^ + 2*F*_c_^2^)/3
2462 reflections	(Δ/σ)_max_ = 0.001
157 parameters	Δρ_max_ = 0.73 e Å^−^^3^
3 restraints	Δρ_min_ = −0.47 e Å^−^^3^

### Special details

**Table:**

Geometry. All e.s.d.'s (except the e.s.d. in the dihedral angle between two l.s. planes) are estimated using the full covariance matrix. The cell e.s.d.'s are taken into account individually in the estimation of e.s.d.'s in distances, angles and torsion angles; correlations between e.s.d.'s in cell parameters are only used when they are defined by crystal symmetry. An approximate (isotropic) treatment of cell e.s.d.'s is used for estimating e.s.d.'s involving l.s. planes.
Refinement. Refinement of *F*^2^ against ALL reflections. The weighted *R*-factor *wR* and goodness of fit *S* are based on *F*^2^, conventional *R*-factors *R* are based on *F*, with *F* set to zero for negative *F*^2^. The threshold expression of *F*^2^ > σ(*F*^2^) is used only for calculating *R*-factors(gt) *etc*. and is not relevant to the choice of reflections for refinement. *R*-factors based on *F*^2^ are statistically about twice as large as those based on *F*, and *R*- factors based on ALL data will be even larger.

### Fractional atomic coordinates and isotropic or equivalent isotropic displacement parameters (Å^2^)

**Table:**

	*x*	*y*	*z*	*U*_iso_*/*U*_eq_	
Cd1	1.0000	0.5000	0.5000	0.03811 (11)	
C1	0.8396 (5)	0.6142 (2)	0.2671 (2)	0.0466 (7)	
H1	0.8465	0.6754	0.3131	0.056*	
C2	0.7823 (5)	0.6301 (2)	0.1550 (2)	0.0456 (7)	
H2	0.7498	0.7002	0.1269	0.055*	
C3	0.7732 (4)	0.5408 (2)	0.0842 (2)	0.0341 (5)	
C4	0.8205 (5)	0.4390 (2)	0.1325 (2)	0.0499 (8)	
H4	0.8146	0.3764	0.0885	0.060*	
C5	0.8761 (5)	0.4304 (3)	0.2458 (2)	0.0497 (7)	
H5	0.9086	0.3612	0.2762	0.060*	
C6	0.7176 (4)	0.5554 (2)	−0.0359 (2)	0.0337 (5)	
C7	0.6323 (5)	0.6241 (3)	−0.1984 (2)	0.0502 (7)	
H7	0.5950	0.6748	−0.2552	0.060*	
C8	0.6534 (5)	0.5154 (3)	−0.2132 (3)	0.0506 (8)	
H8	0.6350	0.4779	−0.2803	0.061*	
N1	0.8860 (4)	0.51635 (18)	0.3142 (2)	0.0396 (5)	
N2	0.6733 (4)	0.6497 (2)	−0.08794 (19)	0.0426 (5)	
N3	0.7075 (4)	0.4715 (2)	−0.1096 (2)	0.0427 (6)	
H3	0.7309	0.4032	−0.0939	0.051*	
N4	0.7084 (3)	0.6785 (2)	0.5340 (2)	0.0411 (5)	
O1	0.8766 (3)	0.6853 (3)	0.5384 (2)	0.0747 (8)	
O2	0.6297 (5)	0.5914 (2)	0.5037 (3)	0.0965 (11)	
O3	0.6156 (3)	0.75586 (18)	0.5590 (2)	0.0562 (6)	
O4	1.2220 (3)	0.63059 (19)	0.4800 (2)	0.0573 (6)	
H4B	1.200 (5)	0.6945 (15)	0.461 (4)	0.086*	
H4A	1.334 (2)	0.617 (3)	0.490 (4)	0.086*	

### Atomic displacement parameters (Å^2^)

**Table:**

	*U*^11^	*U*^22^	*U*^33^	*U*^12^	*U*^13^	*U*^23^
Cd1	0.05052 (19)	0.03477 (16)	0.02528 (16)	−0.00965 (11)	−0.00034 (11)	0.00115 (10)
C1	0.071 (2)	0.0372 (14)	0.0297 (13)	−0.0023 (14)	0.0073 (13)	−0.0033 (11)
C2	0.069 (2)	0.0356 (14)	0.0303 (14)	0.0046 (14)	0.0079 (13)	0.0017 (11)
C3	0.0388 (13)	0.0346 (12)	0.0279 (12)	−0.0030 (11)	0.0050 (10)	0.0008 (10)
C4	0.081 (2)	0.0318 (14)	0.0306 (14)	−0.0012 (14)	−0.0011 (14)	−0.0041 (11)
C5	0.074 (2)	0.0355 (14)	0.0333 (15)	−0.0032 (14)	−0.0040 (13)	0.0036 (12)
C6	0.0389 (13)	0.0332 (13)	0.0285 (12)	0.0001 (10)	0.0059 (10)	−0.0012 (10)
C7	0.065 (2)	0.0547 (18)	0.0284 (14)	0.0112 (15)	0.0049 (13)	0.0079 (12)
C8	0.069 (2)	0.0556 (19)	0.0252 (14)	0.0020 (15)	0.0045 (13)	−0.0020 (12)
N1	0.0523 (14)	0.0368 (12)	0.0267 (12)	−0.0080 (10)	0.0014 (10)	0.0007 (9)
N2	0.0578 (15)	0.0393 (12)	0.0303 (11)	0.0077 (11)	0.0083 (10)	0.0027 (9)
N3	0.0615 (16)	0.0356 (11)	0.0293 (12)	0.0016 (11)	0.0055 (11)	−0.0029 (9)
N4	0.0460 (13)	0.0387 (13)	0.0408 (13)	0.0056 (10)	0.0142 (10)	0.0112 (10)
O1	0.0446 (13)	0.111 (2)	0.0681 (17)	0.0082 (14)	0.0112 (12)	0.0077 (16)
O2	0.114 (3)	0.0367 (14)	0.151 (3)	−0.0131 (15)	0.055 (2)	−0.0218 (17)
O3	0.0654 (14)	0.0389 (11)	0.0700 (16)	0.0107 (11)	0.0271 (12)	0.0021 (10)
O4	0.0418 (11)	0.0386 (12)	0.0886 (18)	−0.0045 (9)	0.0075 (12)	0.0101 (12)

### Geometric parameters (Å, º)

**Table:**

Cd1—N1	2.276 (2)	C5—N1	1.335 (4)
Cd1—N1^i^	2.276 (2)	C5—H5	0.9300
Cd1—O4	2.310 (2)	C6—N2	1.319 (3)
Cd1—O4^i^	2.310 (2)	C6—N3	1.357 (3)
Cd1—O1	2.503 (3)	C7—C8	1.345 (4)
Cd1—O1^i^	2.503 (3)	C7—N2	1.368 (4)
C1—N1	1.333 (4)	C7—H7	0.9300
C1—C2	1.371 (4)	C8—N3	1.364 (4)
C1—H1	0.9300	C8—H8	0.9300
C2—C3	1.386 (4)	N3—H3	0.8600
C2—H2	0.9300	N4—O1	1.211 (3)
C3—C4	1.384 (4)	N4—O2	1.222 (4)
C3—C6	1.463 (3)	N4—O3	1.232 (3)
C4—C5	1.374 (4)	O4—H4B	0.816 (10)
C4—H4	0.9300	O4—H4A	0.814 (10)
			
N1—Cd1—N1^i^	180.00 (4)	N1—C5—C4	123.4 (3)
N1—Cd1—O4	86.81 (9)	N1—C5—H5	118.3
N1^i^—Cd1—O4	93.19 (9)	C4—C5—H5	118.3
N1—Cd1—O4^i^	93.19 (9)	N2—C6—N3	110.6 (2)
N1^i^—Cd1—O4^i^	86.81 (9)	N2—C6—C3	125.8 (2)
O4—Cd1—O4^i^	180.0	N3—C6—C3	123.6 (2)
N1—Cd1—O1	92.58 (9)	C8—C7—N2	110.6 (3)
N1^i^—Cd1—O1	87.42 (9)	C8—C7—H7	124.7
O4—Cd1—O1	71.89 (9)	N2—C7—H7	124.7
O4^i^—Cd1—O1	108.11 (9)	C7—C8—N3	105.9 (3)
N1—Cd1—O1^i^	87.42 (9)	C7—C8—H8	127.1
N1^i^—Cd1—O1^i^	92.58 (9)	N3—C8—H8	127.1
O4—Cd1—O1^i^	108.11 (9)	C1—N1—C5	116.4 (3)
O4^i^—Cd1—O1^i^	71.89 (9)	C1—N1—Cd1	121.42 (18)
O1—Cd1—O1^i^	180.00 (7)	C5—N1—Cd1	121.97 (19)
N1—C1—C2	124.1 (3)	C6—N2—C7	105.5 (2)
N1—C1—H1	118.0	C6—N3—C8	107.4 (3)
C2—C1—H1	118.0	C6—N3—H3	126.3
C1—C2—C3	119.4 (3)	C8—N3—H3	126.3
C1—C2—H2	120.3	O1—N4—O2	118.2 (3)
C3—C2—H2	120.3	O1—N4—O3	122.4 (3)
C4—C3—C2	116.9 (2)	O2—N4—O3	119.4 (3)
C4—C3—C6	122.3 (3)	N4—O1—Cd1	109.1 (2)
C2—C3—C6	120.8 (2)	Cd1—O4—H4B	126 (3)
C5—C4—C3	119.9 (3)	Cd1—O4—H4A	123 (3)
C5—C4—H4	120.0	H4B—O4—H4A	111 (2)
C3—C4—H4	120.0		
			
N1—C1—C2—C3	−0.9 (5)	O1^i^—Cd1—N1—C1	−161.4 (3)
C1—C2—C3—C4	1.1 (5)	N1^i^—Cd1—N1—C5	−114 (15)
C1—C2—C3—C6	−178.3 (3)	O4—Cd1—N1—C5	121.5 (3)
C2—C3—C4—C5	−1.0 (5)	O4^i^—Cd1—N1—C5	−58.5 (3)
C6—C3—C4—C5	178.4 (3)	O1—Cd1—N1—C5	−166.8 (3)
C3—C4—C5—N1	0.7 (6)	O1^i^—Cd1—N1—C5	13.2 (3)
C4—C3—C6—N2	−179.0 (3)	N3—C6—N2—C7	0.5 (3)
C2—C3—C6—N2	0.4 (4)	C3—C6—N2—C7	179.7 (3)
C4—C3—C6—N3	0.1 (4)	C8—C7—N2—C6	−0.6 (4)
C2—C3—C6—N3	179.4 (3)	N2—C6—N3—C8	−0.2 (4)
N2—C7—C8—N3	0.5 (4)	C3—C6—N3—C8	−179.4 (3)
C2—C1—N1—C5	0.6 (5)	C7—C8—N3—C6	−0.2 (4)
C2—C1—N1—Cd1	175.5 (3)	O2—N4—O1—Cd1	−6.3 (4)
C4—C5—N1—C1	−0.5 (5)	O3—N4—O1—Cd1	173.7 (2)
C4—C5—N1—Cd1	−175.3 (3)	N1—Cd1—O1—N4	77.8 (2)
N1^i^—Cd1—N1—C1	72 (15)	N1^i^—Cd1—O1—N4	−102.2 (2)
O4—Cd1—N1—C1	−53.1 (3)	O4—Cd1—O1—N4	163.6 (2)
O4^i^—Cd1—N1—C1	126.9 (3)	O4^i^—Cd1—O1—N4	−16.4 (2)
O1—Cd1—N1—C1	18.6 (3)	O1^i^—Cd1—O1—N4	29 (12)

Symmetry code: (i) −*x*+2, −*y*+1, −*z*+1.

### Hydrogen-bond geometry (Å, º)

**Table:**

*D*—H···*A*	*D*—H	H···*A*	*D*···*A*	*D*—H···*A*
N3—H3···O3^ii^	0.86	2.10	2.923 (3)	160
O4—H4*B*···N2^iii^	0.82 (1)	1.98 (1)	2.796 (3)	174 (4)
O4—H4*A*···O2^iv^	0.81 (1)	2.14 (1)	2.946 (4)	174 (4)
O4—H4*A*···O3^iv^	0.81 (1)	2.65 (3)	3.197 (3)	126 (3)

Symmetry codes: (ii) −*x*+3/2, *y*−1/2, −*z*+1/2; (iii) *x*+1/2, −*y*+3/2, *z*+1/2; (iv) *x*+1, *y*, *z*.
